# Memory-related hippocampal brain-derived neurotrophic factor activation pathways from repetitive transcranial magnetic stimulation in the 3xTg-AD mouse line

**DOI:** 10.1016/j.exger.2023.112323

**Published:** 2023-11-01

**Authors:** M. Windy McNerney, Eric P. Kraybill, Sindhu Narayanan, Fatemeh S. Mojabi, Vaibhavi Venkataramanan, Alesha Heath

**Affiliations:** a Mental Illness Research Education and Clinical Center (MIRECC), Veterans Affairs Palo Alto Health Care System, Palo Alto, CA, USA; b Department of Psychiatry and Behavioral Sciences, Stanford University School of Medicine, Palo Alto, CA, USA; c Medical Anthropology and Global Health, University of Washington, Seattle, WA, USA; d Department of Psychological and Brain Sciences, University of Louisville, Louisville, KY, USA

**Keywords:** Repetitive transcranial magnetic stimulation, Alzheimer’s disease, Brain-derived neurotrophic factor, Object place test, 3xTg-AD, TrkB

## Abstract

Alzheimer’s disease is associated with a loss of plasticity and cognitive functioning. Previous research has shown that repetitive transcranial magnetic stimulation (rTMS) boosts cortical neurotrophic factors, potentially addressing this loss. The current study aimed to expand these findings by measuring brain-derived neurotrophic factor (BDNF), its downstream hippocampal signaling molecules, and behavioral effects of rTMS on the 3xTg-AD mouse line. 3xTg-AD (n = 24) and B6 wild-type controls (n = 26), aged 12 months, were given 14 days of consecutive rTMS at 10 Hz for 10 min. Following treatment, mice underwent a battery of behavioral tests and biochemical analysis of BDNF and its downstream cascades were evaluated via Western blot and ELISA. Results showed that brain stimulation did improve performance on the Object Place Task and increased hippocampal TrkB, ERK, and PLCγ in 3xTg-AD mice with minimal effects on wild-type mice. There was no significant difference in the levels of AKT and Truncated TrkB (TrkB.T1) between treatment and sham. Thus, rTMS has the potential to provide an efficacious non-invasive therapy for the treatment of Alzheimer’s disease through activation of neurotrophic factor signaling.

## 1. Introduction

Repetitive transcranial magnetic stimulation (rTMS) has emerged as a promising means to address the loss of cognitive function associated with Mild Cognitive Impairment (MCI) and Alzheimer’s disease (AD). Current approved treatment options for those suffering from AD are fairly limited and have recently been met with controversy (Ehret and Chamberlin, 2015; Esang and Gupta, 2021). The underlying cause and pathology of this disease have yet to be fully elucidated but researchers are now turning to a plasticity-related explanation rather than focusing on amyloid plaque accumulation (Styr and Slutsky, 2018). As rTMS is thought to noninvasively enhance plasticity in AD and other psychiatric diseases (Li et al., 2021; Cheeran et al., 2008), it is well suited to help alleviate cognitive dysfunction in AD and MCI. At this point, any treatment that could delay the progression of cognitive loss could make a positive impact on the lives of affected individuals and their families.

Although the relationship between rTMS and plasticity has been well-documented, the exact biochemical mechanisms have yet to be thoroughly understood. As plasticity is a biochemically complex process, more research is needed to help uncover the underlying pathways. Brain-derived neurotrophic factor (BDNF) is thought to be a major component in the plasticity-related response to rTMS, which is believed to influence cognition in aged mice (Ma et al., 2014; Ma et al., 2019). Recent research on the 3xTg-AD mouse line showed an increase in cortical BDNF gene expression following rTMS treatment (McNerney et al., 2022). However, an increase in cortical gene expression does not necessarily translate to protein expression and activation of associative downstream molecules. Since AD and MCI are diseases of plasticity, leading to neuronal degeneration predominantly in the hippocampus, we aimed to extend this research by determining if rTMS can increase hippocampal BDNF protein expression and by measuring which downstream pathways are induced.

BDNF binding to the Tyrosine Receptor Kinase B (TrkB) activates at least three arms of downstream signaling kinases and molecules, including Extracellular Signal-Related Kinase (ERK), Protein Kinase B (AKT), and Phospholipase Cγ (PLCγ). While each of these components are involved in TrkB activation, they regulate different plasticity and memory pathways. For example, it is believed that AKT promotes cell survival, ERK leads to cell growth, and PLCγ leads to plasticity (Autry and Monteggia, 2012). In general, each of these many components may be altered in aging and AD and linked to memory dysfunction (Loprinzi et al., 2018). Specifically, research has shown that although aging is not associated with a decrease in TrkB expression, there is an attenuation in the phosphorylation of TrkB and downstream activation of ERK and PLCγ, but not AKT (Cortese et al., 2011). Therefore, in order to understand the therapeutic application of rTMS, it is important to determine the BDNF-TrkB activation pathway.

In addition, there exists an alternatively spliced synaptic form of TrkB, truncated TrkB (TrkB.T1), which increases prevalence with age and degenerative diseases (Fenner, 2012). While full length TrkB (TrkB. FL) promotes the previously described cellular proliferation and survival pathways, TrkB.T1 does not undergo phosphorylation and inhibits the TrkB.FL phosphorylation cascade through a dominant negative mechanism (Carim-Todd et al., 2009). Specifically with AD, beta-amyloid may increase TrkB.T1 receptor expression, and the ratio of these TrkB isoforms is related to spatial memory performance in AD mice (Kemppainen et al., 2012). Thus, exploring the ratio of TrkB.T1 to TrkB.FL in response to rTMS may help uncover a deeper understanding of the relationship between BDNF dependent signaling cascades and neurodegeneration.

Previous treatments targeting BDNF have shown success in animal models. In the APP/PS1 mouse line of AD, AKT and ERK had decreased activation in the hippocampus, while pharmacological treatments to target BDNF only improved ERK activation (Li et al., 2020). Turning to 3xTg-AD mice, treatment with daily chow containing ciliary neurotrophic factor starting at an early age improved memory and increased BDNF and PLCγ expression (Wei et al., 2021). However, in human studies, it is unknown if individuals have AD until well after the onset of pathology. Therefore, treatments need to be devised to modulate BDNF-TrKB signaling later in the lifespan. As cortical BDNF gene expression does increase with rTMS treatment in 12-month-old 3xTg-AD mice, this is a promising avenue that merits further research (McNerney et al., 2022). The effect of BDNF on total TrkB and TrkB.T1, as well as the downstream ERK, AKT, and PLCγ with behavioral performance changes have yet to be fully and systematically investigated with rTMS in animal AD models. Therefore, Western blot analysis from hippocampal homogenates for total TrkB, the TrkB.T1 isoform ratio, ERK, AKT, and PLCγ, along with a suite of behavioral measurements, were conducted following two weeks of daily rTMS in both 3xTg-AD and B6 wild-type mice.

## 2. Materials and methods

### 2.1. Animals

A total of 24 3xTg-AD (Jackson Laboratories-MMRRC) and 26 of their B6129SF2/J wild type control (WT) mice aged 12 months were used in this study. The 3xTg-AD is a transgenic mouse line containing three mutations associated with familial AD, resulting in plaque and tangle pathology, as well as memory deficits. All procedures were carried out under approval from the Institutional Animal Care and Use Committee (IACUC) at the Department of Veterans Affairs, Palo Alto, California. Mice were housed in groups in a temperature and humidity-controlled environment that was maintained on a 12-h light/dark cycle with ad libitum access to food and water. This is the same cohort of mice used in a previous publication that verified the presence of plaques and tangles in the 3xTg-AD mice (McNerney et al., 2022).

### 2.2. rTMS

All mice first underwent surgery to attach a coil support to the skull to allow for rTMS to occur without the need for restraint (Madore et al., 2021). For the surgery, mice were anesthetized with 3 % isoflurane, and a 1–2 cm incision was made into the scalp. The periosteum was scraped away, and the coil support was attached to the skull at a fixed location over the frontal cortex via LockTite glue and dental cement. The scalp was sutured together around the coil support and the mice were allowed to recover for 5 days following surgery. After recovery, mice were habituated to the coil over the course of 3 days. Our rTMS coil is a solenoid composed of copper wire with an outer diameter of 8 mm and an inner diameter of 6 mm. These dimensions allow for the coil to be placed over the coil support and clipped into place for the duration of habituation and stimulation. rTMS followed our previously published methods (McNerney et al., 2022; Madore et al., 2021) and consisted of a square positive pulse at 10 Hz for 10 min, for a total of 6000 pulses, daily for 14 days. The intensity of the stimulation was measured at approximately 20 mT. For the sham condition, mice were treated the same as the active condition, and the coil was attached to the coil support, but no stimulation was administered. The total number of mice used in this study was 52: WT-TMS = 14, WT-SHAM = 12, 3xTg-AD-TMS = 12, 3xTg-AD-SHAM = 12.

### 2.3. Behavior

One day following the 14 days of rTMS, mice underwent 4 days of cognitive behavioral testing. All testing was done starting at 09:00 am. Each day, mice were given 30 min to acclimate to the experimental room, then tested. The experimental area was cleaned with 70 % ethanol between each mouse. On the first day, mice underwent the Open Field Test (OFT) for 10 min in a 50 × 50 cm arena, which also served as the habituation for the Object Recognition Test (ORT). For the OFT, total distance traveled was calculated using automated TopScan software. The following day, mice were exposed to the ORT Sampling Phase with two of the same objects for 10 min. These objects were placed in opposite corners of the square testing area. After 3 h, the mice were exposed to the ORT Test phase for 10 min with one familiar object and one novel object. To determine if exploration of the novel object was greater than the familiar object, we calculated the discrimination index:

$$\frac{\left(\text{novel}\;\text{object}\;\text{exploration})-(\text{familiar}\;\text{object}\;\text{exploration}\right)}{\left(\text{novel}\;\text{object}\;\text{exploration})+(\text{familiar}\;\text{object}\;\text{exploration}\right)}$$

The next day, mice completed the Object Place Test (OPT), which was performed in the exact same manner as the ORT, but with one object being displaced within the area. On the final day, mice completed the Y Maze Spontaneous Alternation Task (Y-Maze) to test working memory. Mice were placed in the center of a three-armed maze, with different symbols at the end of each arm, and allowed to explore for 5 min. Percent alternation was calculated as:

$$\frac{\left(\text{number}\;\text{of}\;\text{entries}\;\text{into}\;\text{arms}\;\text{in}\;\text{sequence}\right)}{\left(\text{total}\;\text{entries}\;\text{into}\;\text{arms}\right)}$$

Time spent exploring objects in the ORT and OPT, and arm entries in the Y-Maze were all scored by experimenters blinded to genotype and treatment.

### 2.4. Biochemical measurements

The day after behavioral testing, we collected vaginal samples to determine the estrus cycle stage of each mouse via vaginal cytology. Then, the mice were perfused with phosphate-buffered saline (PBS) and their brains were extracted. The hippocampus was isolated from the brain, flash frozen on dry ice, and stored at −80 °C until homogenizing. Frozen hippocampi were homogenized on ice with 400 μL cold Syn-Per synaptic Protein Extraction Reagent (Thermo Scientific) containing 1× Protease and Phosphatase inhibitor tablets (Roche). The homogenates were centrifuged at 1200 ×*g* for 10 min and 100 μL of the supernatant was aliquoted and frozen for analysis of the Full Homogenate. The remaining supernatant was re-centrifuged at 15,000 ×*g* for 20 min at 4 °C. This second supernatant is considered the Cytosol Fraction and was separated from the pellet for analysis of downstream protein expression. The pellet, containing the synaptosomes, was resuspended in 100 μL of Syn-Per to create the Synaptic Fraction and was utilized to measure total TrkB (both TrkB.FL and TrkB.T1), TrkB.FL, and TrkB.T1 protein expression. Protein concentrations of all samples were quantified by nanodrop to determine the optimal dilution for biochemical assays.

The Full Fraction was used for BDNF analysis via ELISA in accordance with the instructions provided by the manufacturer (R&D). Samples were diluted 1:10 in PBS, run in duplicate, and quantified by normalization to a Standard Curve from 62.5 to 4000 pg/mL, then normalized to BCA (as measured via the Pierce BCA Protein Assay KIT, Thermo Fisher). Any samples with high variability between duplicates were rerun.

For Western blot, we followed a similar protocol as Heath et al. (Madore et al., 2021). One sample was excluded due to misplacement. Both Cytosolic (ERK, AKT, PLCγ) and Synaptic (TrkB, TrkB.FL, TrkB.T1) homogenates were diluted to 4 μg/mL in tris-buffered saline (TBS) and mixed with 1:4 protein loading buffer and 1:40 mercaptoethanol prior to boiling at 95 °C for 5 min. Samples were then loaded in duplicate into Mini PROTEAN TGX precast gels (BioRad) and separated via electrophoresis at 100 V. Proteins were transferred onto a nitrocellulose membrane (BioRad), washed in the SuperSignal Western Blot Enhancer Pretreatment Solution (Thermo Scientific) and blocked for one hour with Blocking Buffer (Rockland). The membranes were then incubated overnight at room temperature with the following antibodies: TrkB (1:500, BD), ERK1/2 (1:1000 Santa Cruz), AKT (1:500, Cell Signaling), and PLCγ (1:500, R&D). Cofilin (1:2000, Sigma), and β-actin (1:1000, ThermoFisher) were used for the standard loading controls. After rinsing in TBS containing Tween (1 %), the membranes were incubated for 1 h at room temperature in goat anti-rabbit 800 and goat anti-mouse 680 (1:10000, Licor) and imaged using the Odyssey Clx Infrared imaging system (LiCor). Bands were quantified using Gel Analyzer (I L GelAnalyzer, n.d.). TrkB, ERK, AKT, and PLCγ were normalized to their respective loading controls and the isoform TrkB ratio was calculated by dividing the TrkB.T1 by TrkB.FL for each sample.

### 2.5. Statistics

Data were first tested to confirm if they met assumptions of normality as assessed by examining the skewness, kurtosis, and the Shapiro Wilk value of the calculated residuals. There were no violations of homogeneity of variance as assessed using Levene’s statistic. For the analysis of TrkB, assumptions of normality were not met so we used the log transformation of the data which corrected for skewness. For the behavior tests and Western blot data, Two-Way ANOVAs with the Genotype and Treatment as independent variables was performed. For all post-hoc pairwise comparisons, a Tukey adjustment was used with significance at *p <* 0.05. For the exploration of novel and familiar objects, a within groups Repeated Measures Three-Way ANOVA was used, with Genotype and Treatment as independent between-subjects variables and Object Familiarity as the independent within-subjects variable. For post-hoc within-subject comparison of familiar vs novel object, a Sidak adjustment was used with significance at *p <* 0.05. All statistics were performed using SPSS (Version 22, IBM) with graphs produced using GraphPad (Version 7, Prism).

## 3. Results

Analysis of the vaginal histology revealed that there was not a relationship between estrus cycle and genotype (*p* = 0.146) or condition (*p* = 0.827), nor was it related to any of our behavioral and biochemical measures (all *p*’s > 0.05). Thus, the estrus cycle was excluded from our statistical models.

### 3.1. Behavior

**Fig. 1.**
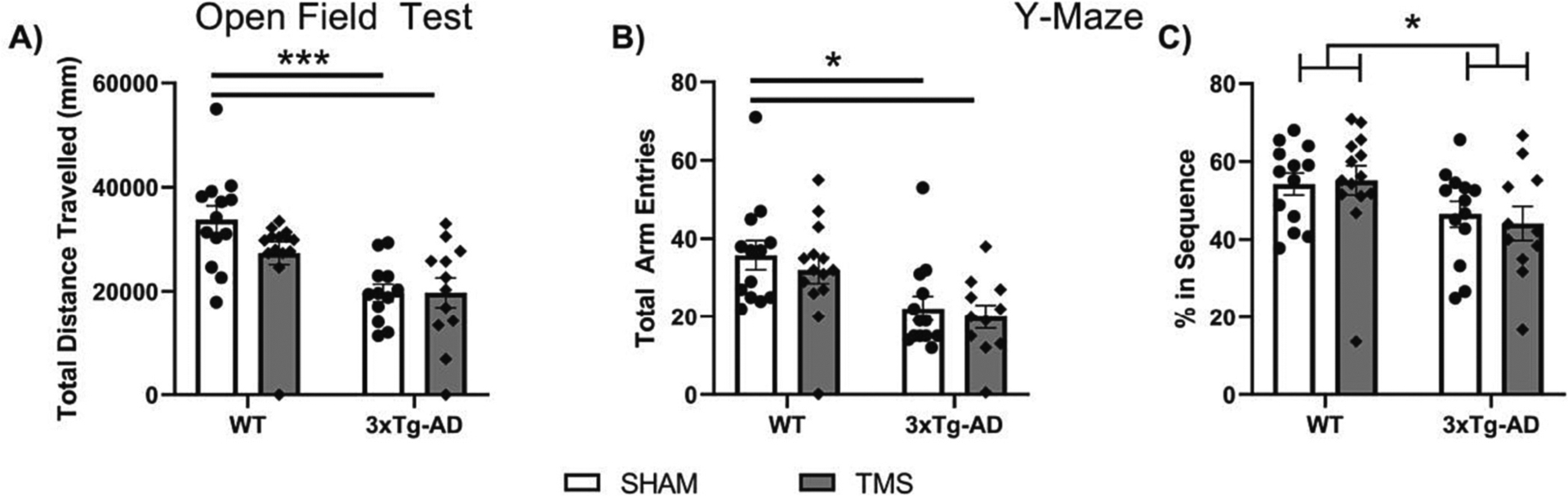
rTMS had no effect on ambulatory differences and working memory in 3xTg-AD mice. Differences in ambulation were measured in 3xTg-AD mice aged 12 months with the A) total distance traveled in the Open Field Test, and B) total arm entries in the Y-Maze. C) Deficits in working memory were apparent in the 3xTg-AD mice using the percent correct entries in sequence in the Y-Maze. rTMS stimulation had no effect on these measures. All figures show mean ± SEM. *p < −0.05, ***p < −0.001.

The OFT was initially performed to assess any ambulatory differences between 3xTg-AD mice and WT controls (Fig. 1A). We did find an overall difference in spontaneous exploratory activity between 3xTg-AD and WT mice (Two Way ANOVA, Effect of Genotype *F*(1,46) = 8.794, *p* = 0.005, $\eta_{p}^{2}=0.160$). There was no effect of rTMS on this measure (Two Way ANOVA, *p >* 0.05).

Working memory was assessed using the Y-Maze (Fig. 1B). For this test, one mouse was removed from analysis in the 3xTg-AD-TMS group as she did not reach at least 10 entries. Overall, the 3xTg-AD mice had fewer entries into the arms of the maze (Two Way ANOVA, *F*(1,45) = 17.852, *p <* 0.001, $\eta_{p}^{2}=0.284$). Even with this effect, the 3xTg-AD mice still had lower percent correct entries (Two Way ANOVA, *F*(1,45) = 12.675, *p* = 0.001, $\eta_{p}^{2}=0.220$), indicating an overall worse working memory (Fig. 1C). There was no effect of rTMS on either of these measures (Two Way ANOVA, *p >* 0.05).

**Fig. 2.**
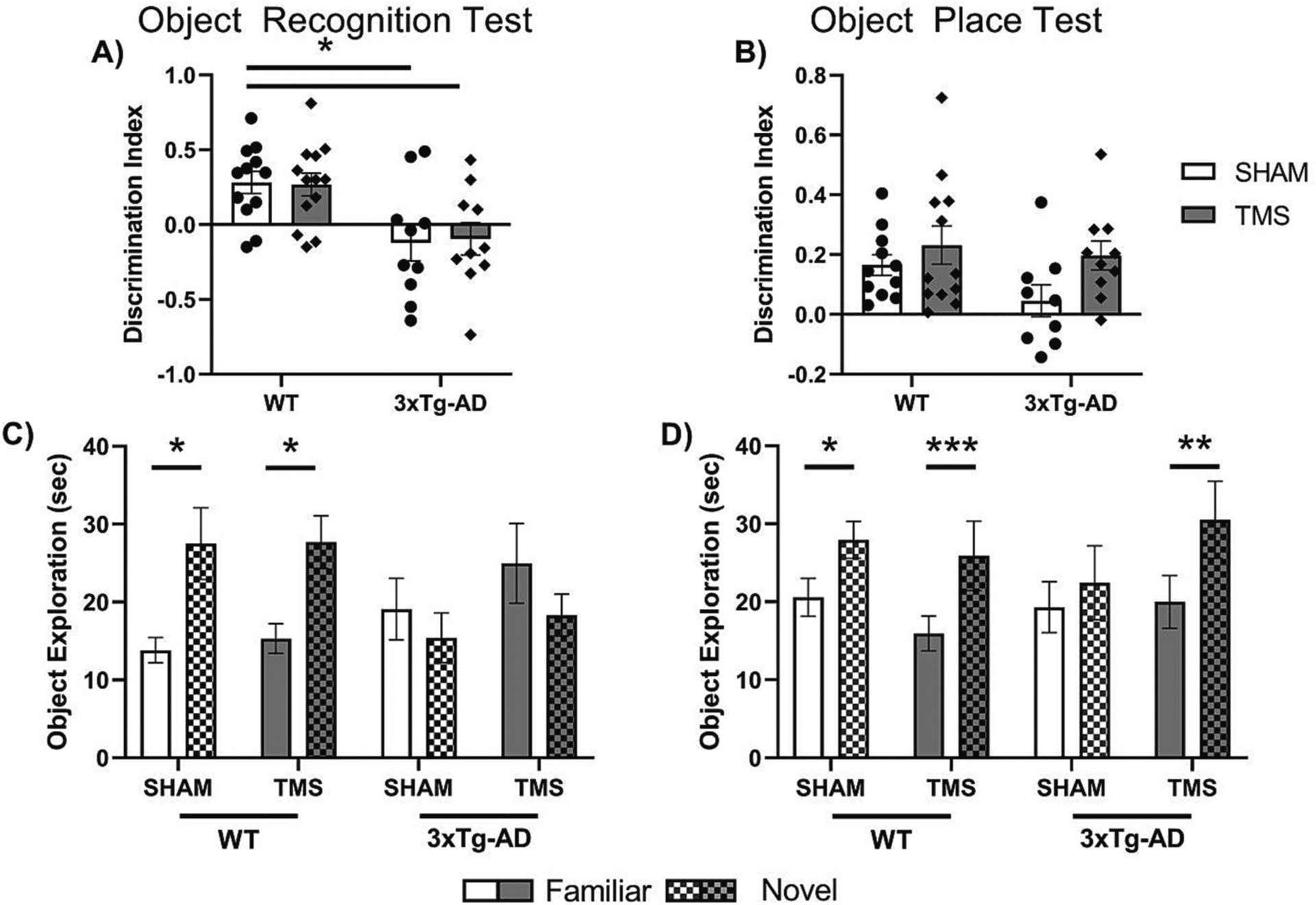
rTMS improved object place memory in 3xTg-AD mice. Discrimination index for A) Object Recognition Test and B) Object Place Test. Within group differences in exploration of the Novel vs Familiar objects for C) Object Recognition Test, and D) Object Place Test. rTMS only improved memory in 3xTg-AD mice in the Object Place Test, there was no effect in the Object Recognition Test. All figures show mean ± SEM. *p < 0.05, **p < 0.01, ***p < 0.001.

Next, the difference in the effect of rTMS on three different components of memory was investigated. Fig. 2 illustrates the results from the ORT and OPT. For both the ORT and the OPT, mice were removed from analysis if they did not reach a total exploration of 20 s in either the familiarization or discrimination phases of the test. For both tests, five mice did not reach this exploration threshold and were excluded from the analysis and for the OPT, an additional three mice did not reach this threshold. This left WT-TMS = 13, WT-SHAM = 12, 3xTg-AD-TMS = 10, and 3xTg-AD-SHAM = 10 used for the analysis of the ORT; and WT-TMS = 12, WT-SHAM = 11, 3xTg-AD-TMS = 10, and 3xTg-AD-SHAM = 9 used for the analysis of the OPT. There was no difference in overall exploration time of the objects in either the OPT or the ORT (Two Way ANOVA, *p <* 0.05, data not shown).

In the ORT, there was a significant effect of genotype on the discrimination index (Two Way ANOVA, *F*(1,41) = 16.672, *p <* 0.001, $\eta_{p}^{2}=0.289$). Post-hoc analysis showed that irrespective of treatment, both 3xTg-AD groups had a significantly lower discrimination index (DI) than WT groups (Tukey post-hoc, *p <* 0.05). In the OPT, there was a significant effect of treatment on the DI (Two Way ANOVA, *F*(1, 38) = 4.345, *p* = 0.0439, $\eta_{p}^{2}=0.103$). Post-hoc analysis showed no individual group differences (Tukey post-hoc, *p >* 0.05).

The difference within each group of exploration of the novel and familiar object was also analyzed to control for within-mouse differences. In the ORT, we found a significant interaction between object and genotype (Three-Way Repeated Measures ANOVA, *F*(1, 41) = 16.225, *p <* 0.001, $\eta_{p}^{2}=0.284$). Post-hoc analysis showed that only the WT groups had a significant difference in novel object exploration (Sidak within subjects post-hoc, *p <* 0.05). In the OPT, there was an overall difference in object exploration (Three-Way Repeated Measures ANOVA, *F*(1, 38) = 36.314, *p <* 0.001, $\eta_{p}^{2}=0.489$) and a mild interaction between object exploration and treatment; however, this did not reach the significance threshold (Three-Way Repeated Measures ANOVA, Effect of Object x Treatment *F*(1, 38) = 3.770, *p* = 0.060, $\eta_{p}^{2}=0.090$). Post-hoc analysis showed that the WT-SHAM, WT-TMS and 3xTg-AD-TMS all had a significant increase in novel object exploration (Sidak within-subjects post-hoc, *p <* 0.05), but not 3xTg-AD-SHAM (Sidak within-subjects post-hoc, *p >* 0.05), indicating that rTMS treatment improved the discrimination of object location in 3xTg-AD but not WT mice.

### 3.2. BDNF, TrkB.FL, and TrkB.T1

**Fig. 3.**
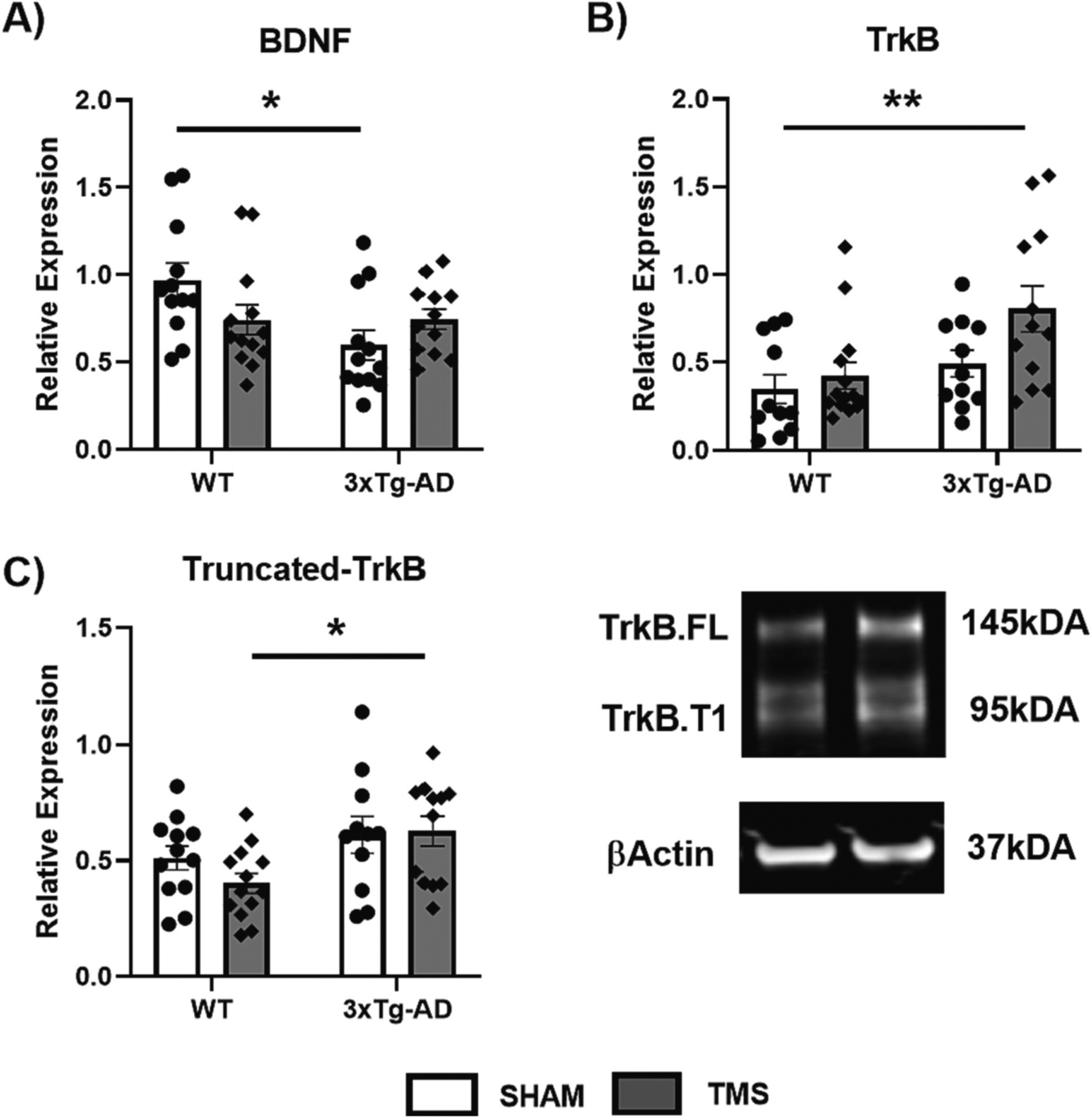
rTMS affects BDNF signaling in 3xTg-AD Mice. Hippocampal synaptic homogenates were used to measure A) brain derived neurotrophic factor (BDNF) using an ELISA, B) Tyrosine Receptor Protein Kinase B (TrkB) normalized to βActin, and C) it’s truncated form (TrkB.T1) normalized to TrkB.FL by Western blot. BDNF was reduced in 3xTg-AD mice which received SHAM stimulation only, and the levels of TrkB and TrkB.T1 expression were increased in 3xTg-AD-TMS mice compared to WT-SHAM and WT-TMS respectively. All figures show mean ± SEM. * *p <* 0.05, ** *p <* 0.01.

Western blot and ELISA were used on the Synaptic homogenates from the hippocampus to determine the effect of rTMS on BDNF and its TrkB receptor isoforms (Fig. 3). There was an overall effect of genotype on the levels of BDNF in the hippocampus (Two Way ANOVA, *F*(1,45) = 4.918, *p* = 0.032, $\eta_{p}^{2}=0.099$). Post hoc analysis showed that 3xTg-AD-SHAM mice had significantly lower BDNF than WT-SHAM mice (Tukey post-hoc, *p <* 0.05). There was also an interaction between treatment and genotype (Two Way ANOVA, *F*(1,45) = 5.086, *p* = 0.029, $\eta_{p}^{2}=0.102$), and although post hoc analysis did not reveal any differences between groups treated with rTMS and SHAM, the 3xTg-AD-TMS group was not statistically different from WT-SHAM (Tukey post-hoc, *p >* 0.05).

For total TrkB levels, there was both an effect of genotype (Two Way ANOVA, *F*(1,44) = 9.750, *p* = 0.003, $\eta_{p}^{2}=0.181$) and treatment (Two Way ANOVA, *F*(1,44) = 4.939, *p* = 0.031, $\eta_{p}^{2}=0.101$). Posthoc analysis revealed the 3xTg-AD-TMS group had elevated levels of TrkB compared to WT-SHAM (Tukey post-hoc *p <* 0.05). For analysis of truncated TrkB isoforms, we analyzed both bands at 95 kDa, for the higher band, which according to the literature may be a summation of TrkB.T-Shc and unglycosylated TrkB.FL (Fleury et al., 2021; Otani et al., 2017). We found no differences amongst groups when normalized to the amount of TrkB.FL (data not shown). For TrkB.T1, the lower band, there was an overall effect of genotype on the ratio of the isoform to TrkB.FL (Two Way ANOVA, *F*(1,44) = 8.201, *p* = 0.006, $\eta_{p}^{2}=0.157$), but overall rTMS had no impact on this ratio (Two Way ANOVA, *p >* 0.05). Between groups there was a greater TrkB.T1 isoform ratio in 3xTg-AD-TMS compared to WT-TMS (Tukey post-hoc *p <* 0.05).

### 3.3. ERK, AKT and PLCγ

**Fig. 4.**
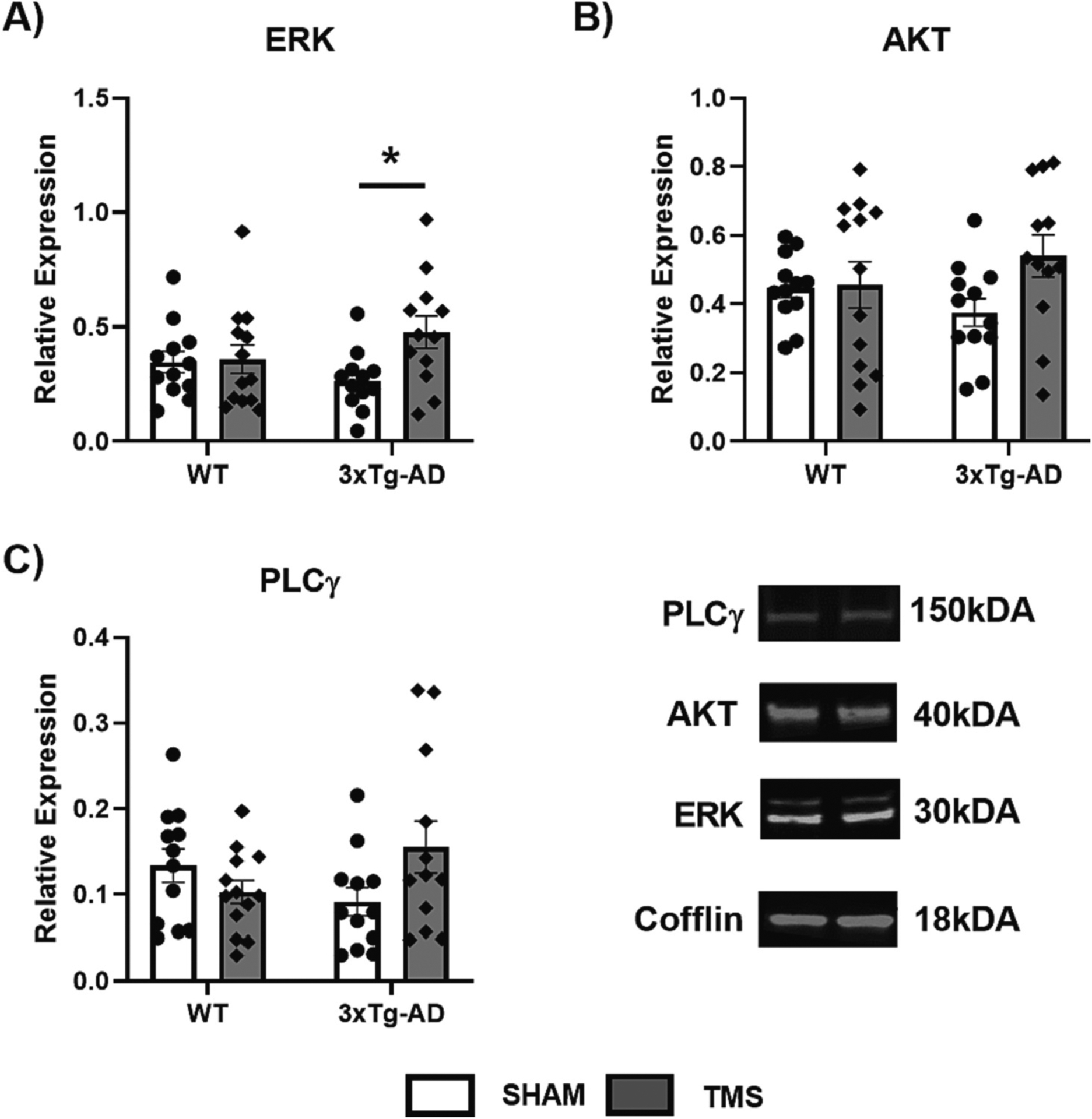
rTMS affects specific downstream targets of BDNF in 3xTg-AD mice. Hippocampal cytosolic homogenates were used to measure downstream targets of BDNF-TrkB signaling. A) Extracellular Signaling-Related Kinase (ERK), B) Protein Kinase B (AKT) and C) Phosphatase Cγ (PLCγ) were all measured using Western blot normalized to Cofflin. rTMS increased the amount of ERK in the 3xTg-AD mice and there was an overall interaction between treatment and genotype in the levels of PLCγ. All figures show mean ± SEM. *p < 0.05.

Western blots on cytosolic homogenates from the hippocampus were assessed to determine the effect of rTMS on the downstream targets of BDNF: ERK, AKT and PLCγ. Fig. 4 illustrates the effects for all three of these measurements. An overall effect of rTMS treatment on the levels of ERK in the hippocampus was observed (Two Way ANOVA, *F*(1,45) = 4.178, *p* = 0.050, $\eta_{p}^{2}=0.083$), with rTMS increasing ERK levels in only the 3xTg-AD mice (Two Way ANOVA, *p <* 0.05). There were no effects of genotype or treatment on AKT levels (Two Way ANOVA, *p >* 0.05). Finally, overall, the levels of PLCγ in the hippocampus were affected by an interaction of both genotype and treatment (Two Way ANOVA, *F* (1,45) = 5.242, *p* = 0.027, $\eta_{p}^{2}=0.104$), but there were no individual differences between groups (Tukey post-hoc, *p >* 0.05).

## 4. Discussion

This study aimed to extend existing literature by measuring the downstream components of rTMS-induced BDNF activation within the hippocampus in addition to assessing memory function. Overall, the results from this study show that rTMS altered a specific component of mouse behavioral performance and different aspects of the BDNF signaling pathway in the 3xTg-AD mouse line. These mice had a deficit in object place performance, object recognition memory, and working memory. rTMS was associated with more exploration time for the location-based memory (OPT). On the other hand, rTMS did not improve location-based memory nor BDNF signaling in our WT cohort. This may be due to the lack of a deficit in these mice, as other research has shown that rTMS can improve spatial memory and hippocampal plasticity in older WT mice (Ma et al., 2019). Additionally, the 3xTg-AD mice had lower BDNF protein levels with a trend toward an increase with rTMS treatment, but heightened TrkB receptor protein expression with treatment. The main downstream response to rTMS was an increase in ERK and PLCγ, both of which are important for plasticity and cellular health (Peng et al., 2010).

In this study, rTMS was only able to improve memory in the object place test. Different brain regions have been shown to be involved in short term object and location recognition memory (Barker and Warburton, 2011). Short term object recognition memory is reliant on the perirhinal cortex, while location memory is dependent on the hippocampus. Therefore, as our rTMS protocol targets superficial layers of the cortex (Madore et al., 2021), specifically focused around the frontal cortex, the effect of stimulation could be targeting downstream areas only relating to location memory. This is in line with some clinical studies investigating the effect of rTMS in MCI which have only shown effects in very specific memory domains. In young healthy WT mice, previous work has shown three days of rTMS was associated with an increase in synaptic connections in both the perirhinal cortex and CA1 regions of the hippocampus needed for long term recognition memory (Heath et al., 2021). The current study utilized 12-month-old WT mice as a control and did not uncover any significant biochemical changes from rTMS in these mice. Including younger and older comparison groups in the future may help further empirically determine the extent at which rTMS can influence biochemical signaling in WT mice. Additionally, future research should investigate if a similar effect of 3 days of rTMS on memory encoding can be found in 3xTg-AD mice, and whether targeting different regions of the brain can influence other memory deficits. Further work is needed to elucidate to the interplay of rTMS and the biochemistry of spatial memory in aged mice and neurodegenerative diseases.

In our 3xTg-AD mice, the deterioration of object recognition memory was greater than that of location memory at 12 months of age. The opposing effect of rTMS on these two forms of memory could also reflect this difference. Currently, there is a clinical consensus that rTMS is more efficacious early in the course of memory loss, possibly when there is still enough healthy underlying tissue to respond to stimulation (Xie et al., 2021). Thus, the object recognition memory loss may require more intensive treatment than the two-week protocol provided in this study; whereas object location memory had a lower deficit to overcome. Consistent with current clinical research, the greatest effects of rTMS are only seen in MCI and early AD patients without severe disease progression (Anderkova and Rektorova, 2014). Our previous work has shown that 6 weeks of rTMS may have greater effects on biological outcomes (McNerney et al., 2022), therefore future research should investigate whether 6 weeks of rTMS (rather than 2 weeks) is better in improving highly deteriorated memory functions.

The data collected showed lower levels of BDNF protein in the hippocampus of the 3xTg-AD mice and not a decrease in TrkB.FL, but rather an increase in TrkB.T1. rTMS elevated overall TrkB levels but had no effect on the isoform ratio. At 12 months of age, our mice do show some pathology, but extensive neuronal pathology is not seen (Belfiore et al., 2019). Therefore, loss of receptor and structural synaptic proteins may be at a minimum and differences in memory are likely derived from changes in intracellular signaling mechanisms, which are influenced by toxic soluble amyloid and calcium dyshomeostasis. Additionally, as BDNF increased only in the 3xTg-AD mice that received treatment, it is expected to see a concurrent increase in TrkB. Thus, the amount of BDNF paired with the corresponding increase in TrkB protein expression may be an important component in optimal BDNF signaling throughout the course of the disease (Hu and Russek, 2008).

rTMS did not have a significant effect on the TrkB isoform ratio. However, the TrkB ratio was significantly higher in 3xTg-AD mice than WT mice, which is consistent with the notion that TrkB.T1 increases with degeneration (Danelon et al., 2016). While it is unclear why rTMS did not alter the isoform ratio, previous research has shown that increasing BDNF does not decrease TrkB.T1 expression (Dorsey et al., 2006). It is possible that longer-term protocols, or a more specific stimulation pattern may be more appropriate to drive expression of specific splice variants (Chiaruttini et al., 2008).

Downstream from BDNF and TrkB, rTMS also elevated ERK and PLCγ pathways, which are primarily involved in synaptic plasticity. Previous work has shown that rTMS increases CREB signaling (Heath et al., 2021). Therefore, inducing protein translation within the neuron causes long lasting changes which can help promote synaptic plasticity. One of the main mechanisms of actions from rTMS is increasing calcium transients within the cell to promote plasticity (Banerjee et al., 2017). The effect of rTMS on PLCγ supports this hypothesis as PLCγ directly activates Inositol Triphosphate (IP3) to release Ca^2+^. Previous research has shown a correspondence between improved behavioral performance and BDNF, along with its downstream pathways in transgenic mice (Wei et al., 2021; Lin et al., 2015).

While ERK is ubiquitous in neural signaling and memory, knockdown models have helped uncover its specific role in mouse behavior. Satoh et al. (2007) discovered that ERK2 is not related to performance on the Y-Maze task, but it was important for long-term spatial memory as measured via the Morris Water Maze task. These findings are well inline with the present data, although our Western antibody covered both ERK1 and ERK2. While systematic investigations of PLCγ in specific memory tests are lacking, it likely plays an important role in both plasticity (Minichiello et al., 2002) and depressive-like behaviors (Rantamäki et al., 2007). Thus, by increasing ERK and PLCγ with rTMS, we expect to see overall improvements in cellular health and memory performance. Future studies should investigate depression and anxiety phenotypes.

There were no significant changes in AKT, but there was a trend for lower AKT in the 3xTg-AD mice. Although our data was associated with some variability, it is possible that a larger sample size may tease out a treatment effect. As AKT is important for cell survival, this effect may play into the fact that rTMS does not appear to be a treatment for reversing neurodegeneration but would better serve to target synaptic plasticity to improve memory. The measurement of AKT in AD mouse lines from previous literature has been inconsistent, with some studies showing no change (Li et al., 2020) and others showing a decrease (Park et al., 2020). Thus, future studies should further investigate the exact relationship between AKT, AD, and BDNF signaling.

## 5. Conclusions

Overall, this study demonstrated that two weeks of daily 10 Hz rTMS improves location-based memory and biochemical signaling in the hippocampus of 3xTg-AD mice. Specifically, rTMS was associated with better discrimination in the OPT, and heightened BDNF-TrkB expression along with downstream ERK and PLCγ. While TrkB.T1 did not respond to 2 weeks of rTMS, its increased level in 3xTg-AD mice compared to WT mice may be helpful in understanding the biology of neurodegeneration. Although object memory performance did not improve, spatial memory and the rate at which object location memory declined did improve. Differences in spatial and object memory improvement may be due to the region targeted for stimulation and the susceptibility of superficial compared to deep brain tissue in relation to rTMS. Spatial memory is an important component in AD-related cognitive deficits (Proskauer Pena et al., 2021), so these results provide promising evidence for rTMS as a non-invasive treatment option for early AD. Even though it is likely that the deficit for the ORT was too far to be overcome, the data lead to the notion that our rTMS parameters may have targeted a specific aspect or circuit of memory. Previous treatments have targeted a single downstream component of TrkB activation; the current regimen recruited two of the three TrkB signaling cascades (Jin, 2020), thus rTMS may be a promising method for inducing neurotrophic factor signaling to help alleviate memory loss.
